# Role of Glycerol-3-Phosphate Dehydrogenase in the Development, Pathogenicity, and Glycerol Biosynthesis of *Aspergillus flavus*

**DOI:** 10.3390/jof12080610

**Published:** 2026-08-14

**Authors:** Liurong Zhang, Jiaru Zhao, Hongyi Lin, Shaoze Wu, Fei Wu, Hongtai Ning, Xiuna Wang, Jun Yuan, Yanling Yang

**Affiliations:** 1 Key Laboratory of Pathogenic Fungi and Mycotoxins of Fujian Province, School of Life Sciences, Fujian Agriculture and Forestry University, Fuzhou 350002, China; 15759021595@163.com (L.Z.); zjrzjr970228@163.com (J.Z.); 18060860625@163.com (H.L.); fafuwsz@163.com (S.W.); 13385565821@163.com (F.W.); 15363386039@163.com (H.N.); xiuna0304@163.com (X.W.); 2 School of Biological Engineering, Jiao Zuo College of Industry and Trade, Jiaozuo 454550, China

**Keywords:** *Aspergillus flavus*, aflatoxin, GfdA, GfdB, glycerol biosynthesis

## Abstract

*Aspergillus flavus*, a ubiquitous phytopathogen, produces mycotoxins, especially aflatoxin B_1_ (AFB_1_), and infects crops worldwide. Glycerol-3-phosphate dehydrogenase (G3PDH) is a key enzyme in the glycerol synthesis and metabolic pathway catalyzing the reversible conversion reaction between glycerol-3-phosphate (G3P) and dihydroxyacetone phosphate (DHAP). However, the biological function of G3PDH in *A. flavus* remains uncharacterized. In this study, the glycerol-3-phosphate dehydrogenase GfdA and GfdB recombinant proteins of *A. flavus* were expressed, and the enzymatic activity of the GfdA protein was successfully determined. Subsequently, single-gene knockout strains (Δ*gfdA*, Δ*gfdB*), double-gene knockout strains (Δ*gfdA*Δ*gfdB*) and their corresponding complemented strains (*gfdA*^C^, *gfdB*^C^) were constructed by a homologous recombination method to explore the biological functions of these two genes in *A. flavus*. The phenotypic analyses revealed that although both *gfdA* and *gfdB* encoded glycerol-3-phosphate dehydrogenases, *gfdA* plays major roles in colony growth, conidiation, sclerotium formation, crop infection and osmotic stress tolerance in *A. flavus*. Notably, the performance of the Δ*gfdA*Δ*gfdB* strains is almost similar to that of the Δ*gfdA* strain. Biochemical assays demonstrated that the intracellular glycerol content increased significantly in all mutants compared to the wild type (WT) under both normal and osmotic stress conditions. Furthermore, we found that exogenous glycerol supplementation rescued the growth defect of the Δ*gfdA* and Δ*gfdA*Δ*gfdB* strains. Taken together, GfdA is important for glycerol synthesis, while GfdB is functionally redundant with respect to GfdA. This study preliminarily explores the main biological functions of GfdA and GfdB, providing a theoretical basis for the study of glycerol anabolic pathways of *A. flavus* and also offering novel insights into the development of strategies to control aflatoxin contamination.

## 1. Introduction

*Aspergillus flavus* is a ubiquitous pathogenic filamentous fungus that is distributed worldwide. It is not only a phytopathogen, but also an opportunistic pathogen in humans and animals [1,2]. On one hand, *A. flavus* can infect corn, peanuts, nuts, soybeans and other crops, causing serious economic losses. On the other hand, the conidia produced by *A. flavus* can easily invade the bodies of humans and animals through multiple routes, such as the respiratory tract, oral cavity, ears, eyes, nasal passages, and skin, causing various diseases and posing a significant threat to public and animal health [3,4]. Furthermore, aflatoxin, the secondary metabolite of *A. flavus*, is recognized as one of the most chemically and physically stable mycotoxins found in nature [5]. To date, over 20 distinct aflatoxins have been identified, and all these variants share a basic structure characterized by the presence of a difuran ring and a coumarin moiety (oxaphthalene ketone) [6]. The International Agency for Research on Cancer (IARC) has classified aflatoxin as a Group I carcinogen [7]. Among them, aflatoxin B_1_ (AFB_1_) is notorious for its highest production, extreme toxicity, and potent carcinogenicity.

Glycerol is the simplest trihydric alcohol, and there is a relatively complete and uncomplicated glycerol anabolic pathway in eukaryotic cells. The primary reason for the production of glycerol by eukaryotic cells is that glycerol can respond to external stress, especially hypertonic stress, oxidative stress and heat stress [8]. And also, glycerol can be used as an alternative carbon source. Previous studies have reported that in the model fungus *Saccharomyces cerevisiae*, glycerol synthesis begins with the reduction of DHAP to G3P by NADH-dependent glycerol-3-phosphate dehydrogenase (GPD) [9]. Subsequently, G3P is dephosphorylated to glycerol by glycerol-3-phosphate phosphatase (GPP). Nevertheless, in the model filamentous fungus *Aspergillus nidulans*, it has also been proposed that glycerol was produced by using dihydroxyacetone (DHA) as an intermediate. In this pathway, the glycolytic intermediate DHAP is dephosphorylated to DHA, which is then reduced to glycerol [10].

G3PDH is a key enzyme in the glycerol synthesis pathway and can catalyze the production of G3P from DHAP [9,11]. Most bacterial and mammalian genomes encode only GAPDH, whereas fungal and plant genomes encode many GAPDH isozymes. *Arabidopsis thaliana* has seven genes that encode cytosolic NAD^+^-dependent GAPDH and chloroplast NADP^+^-dependent GAPDH [12]. For *S. cerevisiae* cells, there are two isoenzymes of GPD (GPD1 and GPD2). Previous studies have shown that GPD1 is a target gene of the HOG pathway and its expression is induced by osmotic pressure [13,14]. However, GPD2 is induced when cells are in an anaerobic environment [15]. Some studies have shown that GPD1 is located in organelles in the cytoplasm, with a small part present in peroxisomes, while GPD2 is also mainly located in the cytoplasm, with a few in mitochondria [16]. Some studies have found that in *S. cerevisiae*, the *gpd1* and *gpd2* genes have different effects on the synthesis of glycerol in the strain. Compared to the wild type, the *Gpd1* deletion mutant strain has significantly reduced or even completely absent glycerol synthesis ability [11,17,18]. *Gpd1*-overexpressing strain can significantly increase the intracellular glycerol content [19], but the *Gpd2* deletion mutant strain showed no significant difference in intracellular glycerol content compared with the wild type [15]. Recent studies have shown that in *S. cerevisiae*, Hog1 regulates glycerol synthesis by relieving Ypk1-mediated inhibition of Gpd1 [20]. In *A. nidulans*, glycerol-3-phosphate dehydrogenase Gfd, which can catalyze the production of G3P from DHAP, is located in the cytoplasm and can bind to the coenzyme NAD^+^. The study confirmed that both Gfd isozymes contribute to the environmental stress defense of *A. nidulans*, but their functions are specific, with GfdA involved in osmotic stress defense and GfdB involved in oxidative stress defenses in *A. nidulans* [20,21]. GfdA had a limited effect on glycerol synthesis, and glycerol accumulation was not reduced during conidial germination, but intracellular glycerol levels were reduced to 60% of wild type during vegetative growth under osmotic stress conditions [21]. It was found that the introduction of the *gfdB* gene from *A. nidulans* into *Aspergillus glaucus* results in an enhanced ability of GfdB to improve multi-stress tolerance in the heterologous host [20]. In subsequent experiments, the introduction of *gfdB* into *Aspergillus wentii* conferred only a modest enhancement of stress tolerance and partially reversed the osmophilic phenotype of the recipient strain [22]. This also indicated that even between closely related species, the function of GfdB may differ. Studies also indicated that the gene *gfdA* encoding G3PDH of *A. fumigatus* is essential for normal colony growth in glucose medium [23]. Furthermore, overexpression of the *GfdA* homolog GfdB failed to rescue the phenotype of the GfdA deletion mutant, suggesting that GfdA plays an important role in the synthesis of G3P and glycerol. Overexpression of the gene encoding the predicted glycerol kinase GlcA, capable of phosphorylating glycerol to form G3P, was able to compensate for the growth defect of the *GfdA* deletion mutant, suggesting that the growth defect of *GfdA* deletion mutants may be due to the absence of G3P rather than glycerol [23].

However, the functional roles of the genes encoding glycerol-3-phosphate dehydrogenase in *A. flavus* remain largely unexplored. In this study, we therefore set out to investigate the enzymatic characteristics of GfdA and GfdB, their respective contributions to fungal growth, development, and stress adaptation, and their potential link to aflatoxin accumulation. Elucidation of these questions will not only deepen our understanding of glycerol metabolism in *A. flavus*, but may also inform novel strategies for aflatoxin contamination control.

## 2. Materials and Methods

### 2.1. Strains and Culture Conditions

All strains studied are listed in Table 1. All strains were cultured on YES and PDA media for vegetative growth and conidiation, as well as on CM medium for sclerotia formation. YES liquid medium was used for aflatoxin production as described before [24].

### 2.2. Phylogenetic Analysis

The protein sequences of *A. flavus* GfdA (QRD87930.1) and GfdB (XP_041150538.1) were retrieved from the NCBI (National Center for Biotechnology Information, United States) database and compared with the corresponding sequences of *A. nidulans*, *Aspergillus niger*, *Aspergillus oryzae*, *Aspergillus fumigatus*, *Saccharomyces pombe* and *S. cerevisiae* via BLASTP 2.17.0+ alignment. A maximum likelihood tree was generated using MEGA-7.0 software with a JTT Matrix model and 1000 bootstrap replicates. Protein domains were predicted by SMART (http://smart.embl-heidelberg.de/, accessed on 1 April 2026) software and drawn by DOG 2.0 software.

### 2.3. Expression of Recombinant Protein and Assay of Enzyme Activity

All *E. coil* strains and plasmid are listed in Table 2. The *gfdA* gene was cloned by PCR using special primers that included restriction sites for *BamH*I and *Xho*I. The pET32a(+) vector and *gfdA* gene fragment were digested with *BamH*I and *Xho*I and then ligated by *Exo*III. The pET32a(+) vector containing the *gfdA* gene fragment was transformed into an expression strain of *E. coli* BL21 grown in liquid LB medium and further incubated at 37 °C until the OD value reached 0.5. Isopropyl-β-D-thiolactopyranoside (0.4 mM) was added to the medium to induce target protein expression. After 18 h of induction, cells were harvested and sonicated on ice, and then Ni^2+^-NTA column was used for purification. The expression of recombinant protein GfdB was carried out by pET28a(+) vector. The enzymatic activity was determined according to the method of Ghoshal et al. [25]. The absorbance at 340 nm was measured with a microplate reader and plotted as a graph.

### 2.4. Construction of Knockout and Complemented Mutants

Construction of knockout and complemented mutants was carried out by homologous recombination [26]. Table 3 lists the primers required for this study. For the construction of knockout mutants, the fusion product (5′-UTR-*pyrG*-3′-UTR) was amplified by PCR and transformed into protoplasts of *A. flavus* CA14. Correct knockout transformants were identified by PCR: the ORF fragment of the target gene could not be amplified, whereas the 5′-UTR-*pyrG* (AP) and pyrG-3′-UTR (BP) fragments indicative of proper homologous recombination were successfully amplified. Furthermore, qRT-PCR was performed to confirm the loss of target gene expression in the knockout strains. Double-gene knockout mutants were constructed based on single knockout mutants, using the selectable marker gene *ptrA* with pyrithione resistance to replace a second target gene, with the same PCR and qRT-PCR confirmation strategy applied.

For the construction of complementation strains, the 5′-UTR-target gene-*ptrA*-3′-UTR fragment was introduced into protoplasts of the corresponding single knockout strains to replace the *pyrG* gene, followed by selection with pyrithione. The 5′-UTR-ORF (AP) and *ptrA*-*pyrG* (BP) fragments indicative of proper homologous recombination were successfully amplified. Then qRT-PCR was used to confirm restoration of target gene expression. All primer pairs used for validation are listed in Table 3.

### 2.5. Analysis of Colony Growth, Conidiation and Sclerotia Formation

To record colony growth, 1 μL of conidia suspensions (1 × 10^6^ pcs/mL) of different strains were inoculated into YES and PDA media, and diameters were measured 3 d later. For conidial counting, 1 μL of the conidia suspension was inoculated on PDA medium and incubated at 37 °C for 3 d. Then the conidia were washed down with 3 mL of distilled water and then counted by a hemocytometer. To determine the production of sclerotia, 1 μL of the conidia suspensions of each strain were incubated at 37 °C for 7 d, and then the conidia were washed away with 75% ethanol to count the sclerotia [27].

### 2.6. Aflatoxin Analysis

The aflatoxins were extracted with dichloromethane and then transferred to a centrifuge tube and evaporated to dryness. Aflatoxin production was detected by thin-layer chromatography (TLC), and the results were observed under 365 nm UV light. The JD-801 computer-aided image analysis system (JEDA Co., Nanjing, China) was used for quantitative analysis of aflatoxin biosynthesis [28].

### 2.7. The Infection of Peanuts and Corn Seeds

To test the infectivity of the strains, conidia suspensions of different strains were inoculated onto the surface of peanuts and corn, which were pretreated according to the method of Yang et al. [27]. The inoculated seeds were then placed in a Petri dish covered with wet filter paper and incubated at 29 °C in the dark for 5 d. Then conidia were counted and aflatoxin was analyzed as described above.

### 2.8. Determination of Intracellular Glycerol Content of A. flavus

The intracellular glycerol content of *A. flavus* was quantified using a Tissue Cell Glycerol Enzymatic Assay Kit (Applygen, Beijing, China). Briefly, conidial suspensions from different strains were inoculated into the culture medium at equal densities; after 48 h, mycelia were harvested, scraped, and freeze-dried with liquid nitrogen. Subsequently, the samples were weighed, lysed, and analyzed for glycerol content according to the manufacturer’s instructions, with absorbance measured at 550 nm [29].

### 2.9. Quantitative RT-PCR

The mycelia of the strains were grown on YES medium at 37 °C. Total RNA from mycelium was extracted with a RNA Extraction Kit (Tianmo Bio, Jiangsu, China), and reverse transcribed cDNA was obtained by TransScript one-step gDNA Removal and cDNA Synthesis SuperMix (TransGen Biotech, Beijing, China) [30]. SYBR Green qPCR Mix (Guangzhou Dongsheng Biotech, Guangzhou, China) was used for qRT-PCR on a Thermo Fisher Scientific real-time PCR system (Vantaa, Finland). The relative expressions of the target genes were calculated using the *actin* gene of *A. flavus* as the reference [31]. The primers for qRT-PCR are listed in Table 3.

### 2.10. Statistical Analysis

Statistical analyses were performed using GraphPad Prism 6. A *p*-value < 0.05 was considered statistically significant. The T-test was applied for pairwise comparisons, and analysis of variance (ANOVA) was used for multiple group comparisons.

## 3. Results

### 3.1. Identification and Analysis of GfdA and GfdB in A. flavus

**Figure 1:**
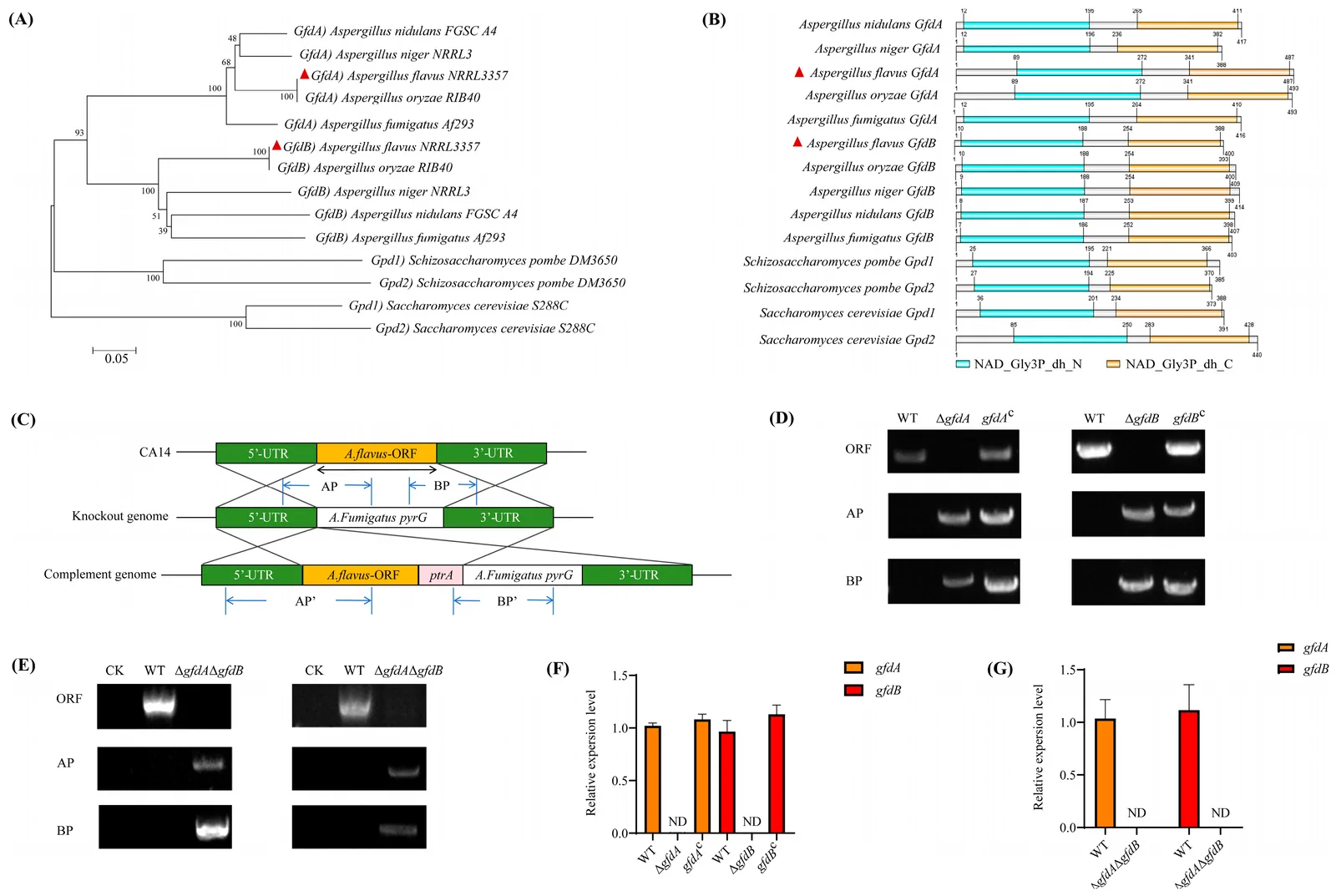
Phylogentic analysis of GfdA, GfdB and mutant strains construction. (**A**) The phylogenetic tree of GfdA and GfdB proteins from different species. The phylogenetic tree was constructed using MEGA 7.0 software and the neighbor-joining method with 1000 replications. Protein ID numbers from NCBI are as follows: AflGfdA (XP_002373049.1, *Aspergillus flavus NRRL 3357*), AnGfdA (CAK37086.1, *Aspergillus niger NRRL3*), AnGfdA (XP_657955.1, *Aspergillus nidulans FCSG A4*), AoGfdA (XP_001817892.3, *Aspergillus oryzae RIB40*), AfGfdA (XP_749965.1, *Aspergillus fumigatus Af293*), SpGpd1 (NP_596682.1, *Saccharomyces pombe DM3650*), ScGpd1 (NP_010262.1, *Saccharomyces cerevisiae S288C*), AflGfdB (XP_002378138.1, *Aspergillus flavus NRRL 3357*), AnGfdB (CAK48573.1, *Aspergillus niger NRRL3*), AnGfdB (EAA58610.1, *Aspergillus nidulans FCSG A4*), AoGfdB (XP_001826450.1, *Aspergillus oryzae RIB40*), AfGfdB (XP_755159.2 *Aspergillus fumigatus Af293*), SpGpd2 (NP_594542.1, *Saccharomyces pombe DM3650*), ScGpd2 (NP_014582.1, *Saccharomyces cerevisiae S288C*). (**B**) Comparison of the conserved NAD_Gly3P_dh_N and NAD_Gly3P_dh_C domains of GfdA and GfdB proteins across different species. The domains of the above protein were predicted on the Simple Modular Architecture Research Tool (SMART: http://smart.emblheidelberg.de/, accessed on 1 April 2026) website, and finally the DOG 2.0 software was used to create a domain visualization diagram. (**C**) Scheme for gene deletion and complementation strains construction using a homologous recombination method. (**D**,**E**) PCR verification of WT, Δ*gfdA*, *gfdA^C^*, Δ*gfdB*, *gfdB^C^* and Δ*gfdA*Δ*gfdB* strains. gDNA was used as a template. AP and BP can be amplified in positive transformants of all strains, while ORF can only be amplified in WT. (**F**,**G**) The expression level of *gfdA* and *gfdB* genes in all strains. ND represents Not Detected.

In order to explore the evolutionary relationship of glycerol-3-phosphate dehydrogenase in different species, the protein sequences of GfdA and GfdB in *A. flavus* were retrieved from the NCBI database. And then Blastp searches were conducted to compare with other *Aspergillus* and yeast proteins, such as *A. fumigatus*, *A. nidulans*, and *S. cerevisiae*, etc. A phylogenetic tree was constructed by MEGA 7.0 software. The analysis revealed that GfdA and GfdB proteins in the *Aspergillus* genus are highly conserved, but there are still evolutionary differences from yeast (Figure 1A). Subsequently, protein domains were analyzed by DOG 2.0. The results showed that all examined proteins have two coenzyme-binding conserved domains: an N-terminal NAD-Gly3P-dh-N domain and a C-terminal NAD-Gly3P-dh-C domain (Figure 1B). To further elucidate the function of GfdA and GfdB, gene knockout strains (Δ*gfdA*, Δ*gfdB*), complementary strains (*gfdA^C^*, *gfdB^C^*) and double knockout strain (Δ*gfdA*Δ*gfdB*) were constructed using a homology rearrangement strategy (Figure 1C). The positive strains were verified with a PCR method (Figure 1D,E) and qRT-PCR (Figure 1F,G), and the results showed that all mutant strains were successfully constructed for further function study.

### 3.2. Recombinant Expression and Enzyme Activity Assay of GfdA and GfdB Proteins

**Figure 2:**
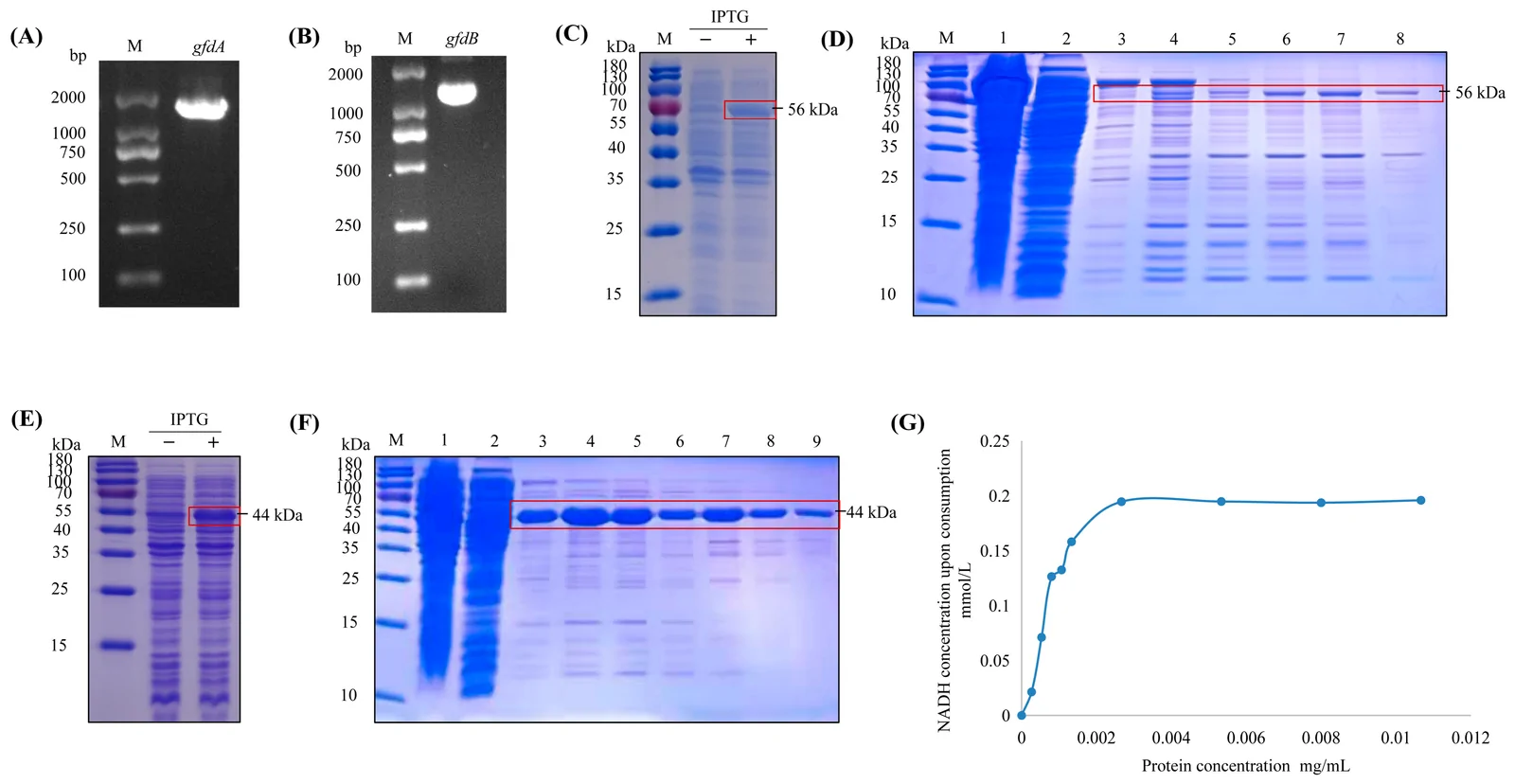
The enzyme activities of GfdA and GfdB were verified in vitro. (**A**,**B**) PCR results of *gfdA* and *gfdB* genes amplificated by cDNA. (**C**) Expression level of GfdA protein was detected by SDS-PAGE gel electrophoresis. Obvious overexpression was detected in the vicinity of the 55–70 kDa molecular weight marker when IPTG was added. (**D**) The GfdA protein was purified using nickel-affinity chromatography. Lane 1, pellet; Lane 2, flow-through from the nickel column; Lanes 3–8, proteins eluted with buffers of varying concentrations (pH 7.4). (**E**) Expression level of GfdB protein was detected by SDS-PAGE gel electrophoresis. Obvious overexpression was detected in the vicinity of the 40–55 kDa molecular weight marker when IPTG was added. (**F**) The GfdB protein was purified using nickel-affinity chromatography. Lane 1, pellet; Lane 2, flow-through from the nickel column; Lanes 3–9, proteins eluted with buffers of varying concentrations (pH 8.0). (**G**) In vitro NADH-dependent enzymatic activity of the GfdA protein.

To determine whether *A. flavus* GfdA and GfdB proteins have glycerol-3-phosphate dehydrogenase activity, the coding sequences of GfdA and GfdB proteins were cloned using the cDNA of *A. flavus* WT as a template (Figure 2A,B) and ligated into pET32a(+) and pET28a(+) vectors, respectively. After sequencing and secondary transformation, the recombinant expression strains were successfully constructed. Subsequently, recombinant protein expression was induced by IPTG, and the target proteins were purified using Ni^2+^-NTA affinity chromatography, yielding GfdA protein (Figure 2C,D). The same procedure was applied to obtain GfdB protein (Figure 2E,F). Finally, an in vitro enzymatic assay demonstrated that GfdA protein possessed glycerol-3-phosphate dehydrogenase activity (Figure 2G), whereas no such activity was detected for GfdB in this study.

### 3.3. Effects of gfdA and gfdB Genes on the Growth and Conidiation of A. flavus

**Figure 3:**
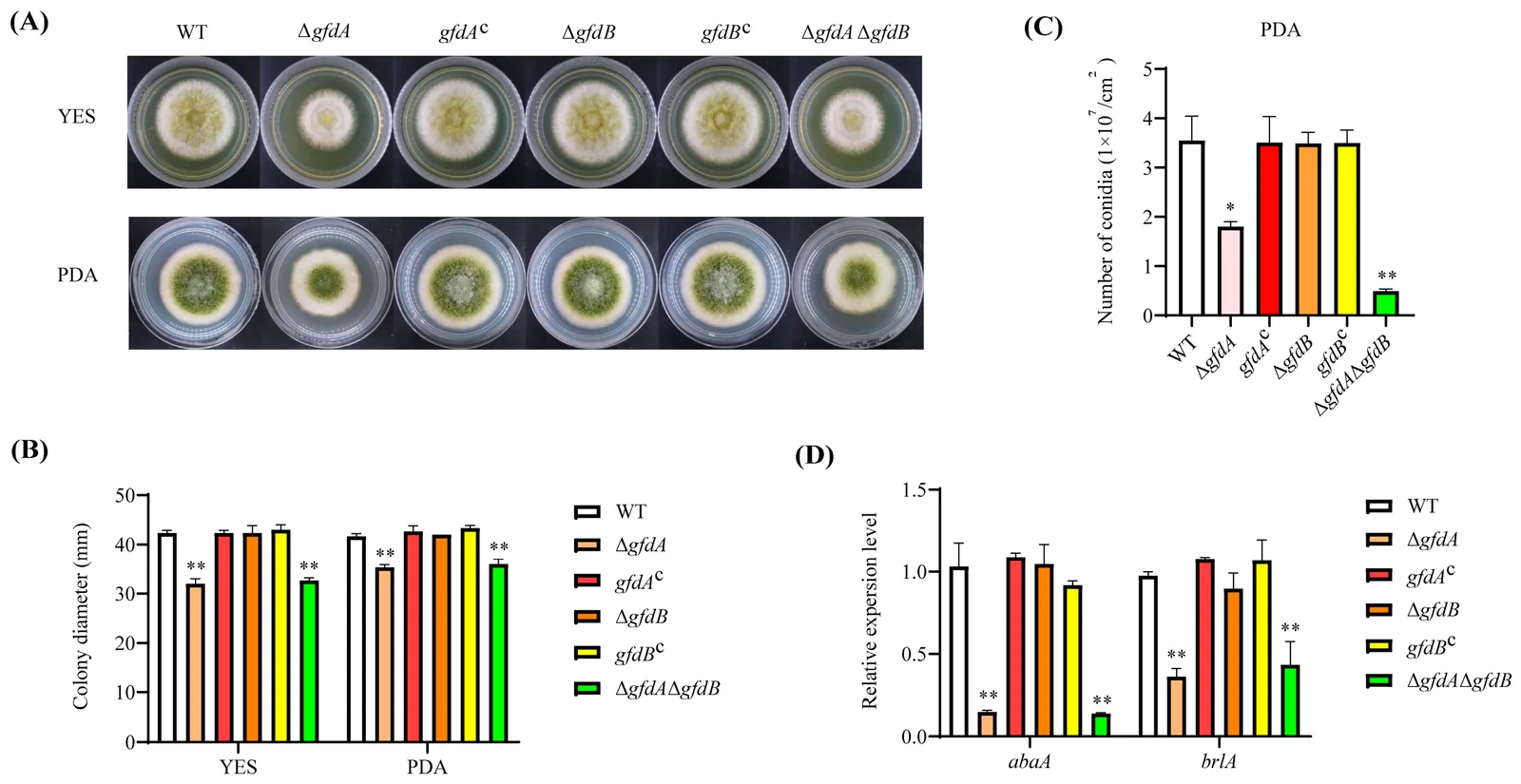
Effects of *gfdA* and *gfdB* on growth and development of *A. flavus*. (**A**) The phenotype of the WT, Δ*gfdA*, Δ*gfdB*, *gfdA*^C^, *gfdB*^C^ and Δ*gfdA*Δ*gfdB* strains cultured on YES and PDA solid media for 3 d at 37 °C in dark conditions. (**B**) Colony diameter of all strains on YES and PDA solid media. (**C**) The number of conidia produced by all strains on YES and PDA solid media. (**D**) Relative expression levels of *abaA* and *brlA* in all strains. The primers used for RT-qPCR are shown in Table 3. The asterisks in (**B**–**D**) represent significant difference levels when Δ*gfdA*, Δ*gfdB* and Δ*gfdA*Δ*gfdB* strains were compared with WT (i.e., * *p* ≤ 0.05, ** *p* ≤ 0.01). Standard deviation is indicated by error bars.

To investigate whether *gfdA* and *gfdB* genes play a role in the growth of *A. flavus*, quantitative conidia suspensions of different strains were inoculated on YES and PDA solid medium and cultured at 37 °C for 3 d in the dark (Figure 3A). It was observed that the colony diameter of the Δ*gfdA* and Δ*gfdA*Δ*gfdB* strains decreased and the colony edge appeared irregular, while the Δ*gfdB* strain did not change, compared with the wild type (WT) (Figure 3B). The results indicated that GfdA exerts a regulatory effect on the growth of *A. flavus*, while GfdB has no obvious impact on its growth. After growing on PDA plates for 4 d, compared to WT, the conidia production of Δ*gfdA* and Δ*gfdA*Δ*gfdB* strains was significantly reduced, while the Δ*gfdB* strain did not change (Figure 3C). The results revealed that the *gfdA* gene was involved in the regulation of conidiation in *A. flavus*, but the *gfdB* gene was not. In fungi, the formation of conidia is mainly regulated by *abaA* and *brlA* genes [32,33], so this study also detected the expression levels of these genes at the transcriptional level. It was discovered that the expression levels of *abaA* and *brlA* genes were significantly downregulated in the Δ*gfdA* and Δ*gfdA*Δ*gfdB* strains, but did not change in Δ*gfdB*, when compared with WT (Figure 3D). Together, it can be seen that the Δ*gfdA* and Δ*gfdA*Δ*gfdB* strains regulate the formation of conidia by regulating the *abaA* and *brlA* genes.

### 3.4. Involvement of gfdA and gfdB Genes in Sclerotium Formation of A. flavus

**Figure 4:**
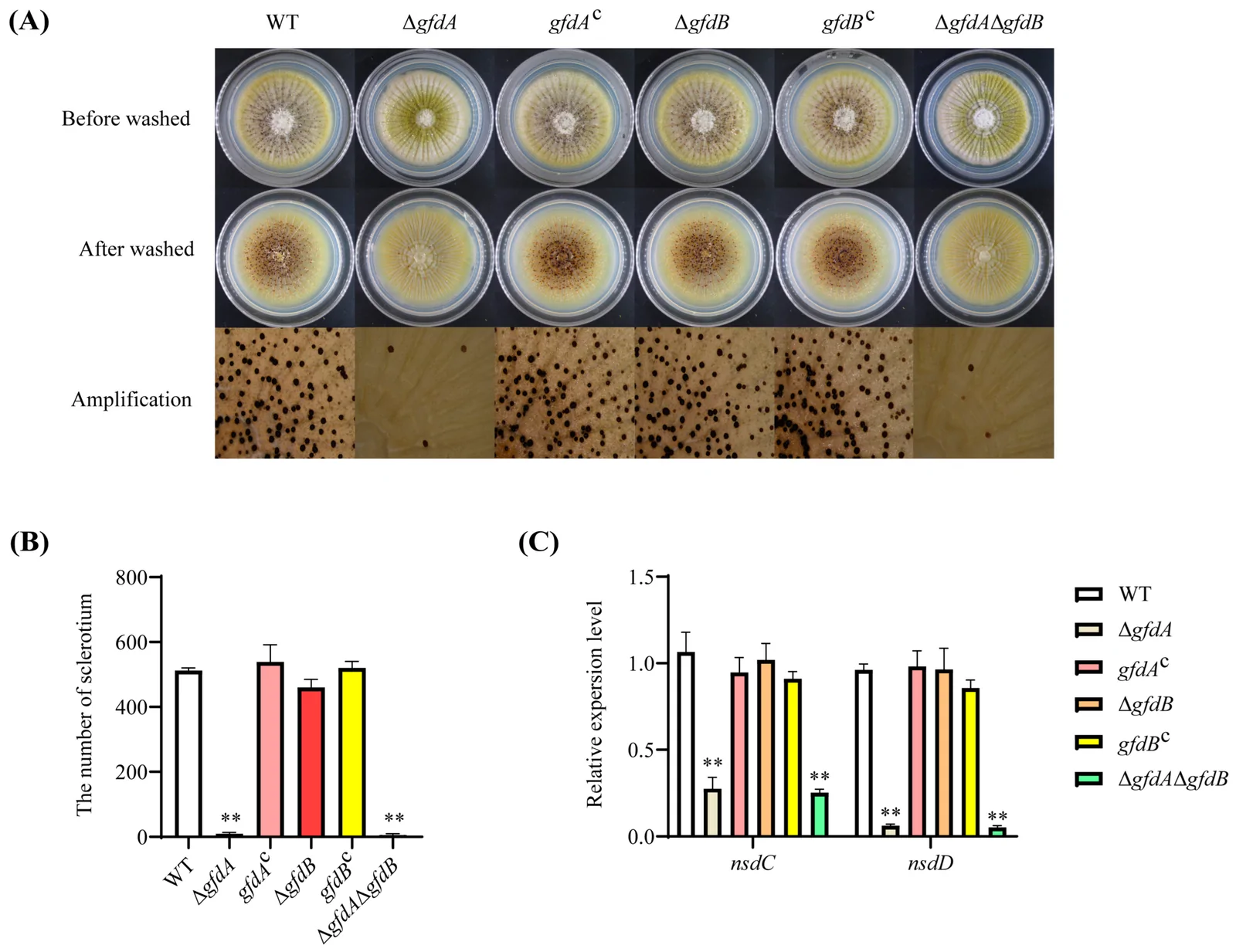
Effects of *gfdA* and *gfdB* on sclerotium formation of *A. flavus*. (**A**) The phenotype of the WT, Δ*gfdA*, Δ*gfdB*, *gfdA*^C^, *gfdB*^C^ and Δ*gfdA*Δ*gfdB* strains cultured on the CM medium for 7 days at 37 °C in dark conditions. (**B**) Quantification of sclerotia of all strains in (**A**). (**C**) Relative expression levels of *nsdC* and *nsdD* genes in all strains. The primers used for RT-qPCR are shown in Table 3. The asterisks in (**B**,**C**) represent significant difference levels when Δ*gfdA*, Δ*gfdB* and Δ*gfdA*Δ*gfdB* strains were compared with WT (i.e., ** *p* ≤ 0.01). Standard deviation is indicated by error bars.

To study the effects of the gfdA and gfdB genes on sclerotium formation of *A. flavus*, conidia suspensions of strains were inoculated into CM solid medium and cultured at 37 °C for 7 d in the dark (Figure 4A). The sclerotia were then counted, and it indicated that compared to WT, the sclerotium production of the Δ*gfdA* and Δ*gfdA*Δ*gfdB* strains was significantly reduced, while the Δ*gfdB* strain did not change obviously (Figure 4B). As previously reported, sclerotium morphogenesis in fungi is mainly regulated by the *nsdC* and *nsdD* genes [34]. To further dissect how GfdA and GfdB regulate the sclerotium formation in *A. flavus*, the transcriptional level of the *nsdC* and *nsdD* genes was detected. Compared to WT, the expression of Δ*gfdA* and Δ*gfdA*Δ*gfdB* was obviously downregulated, while that of Δ*gfdB* was not changed (Figure 4C), suggesting that GfdA affected the expression levels of *nsdC* and *nsdD* genes, thereby influencing the sclerotium production of *A. flavus*.

### 3.5. Effects of gfdA and gfdB Mutations on Aflatoxin Biosynthesis

**Figure 5:**
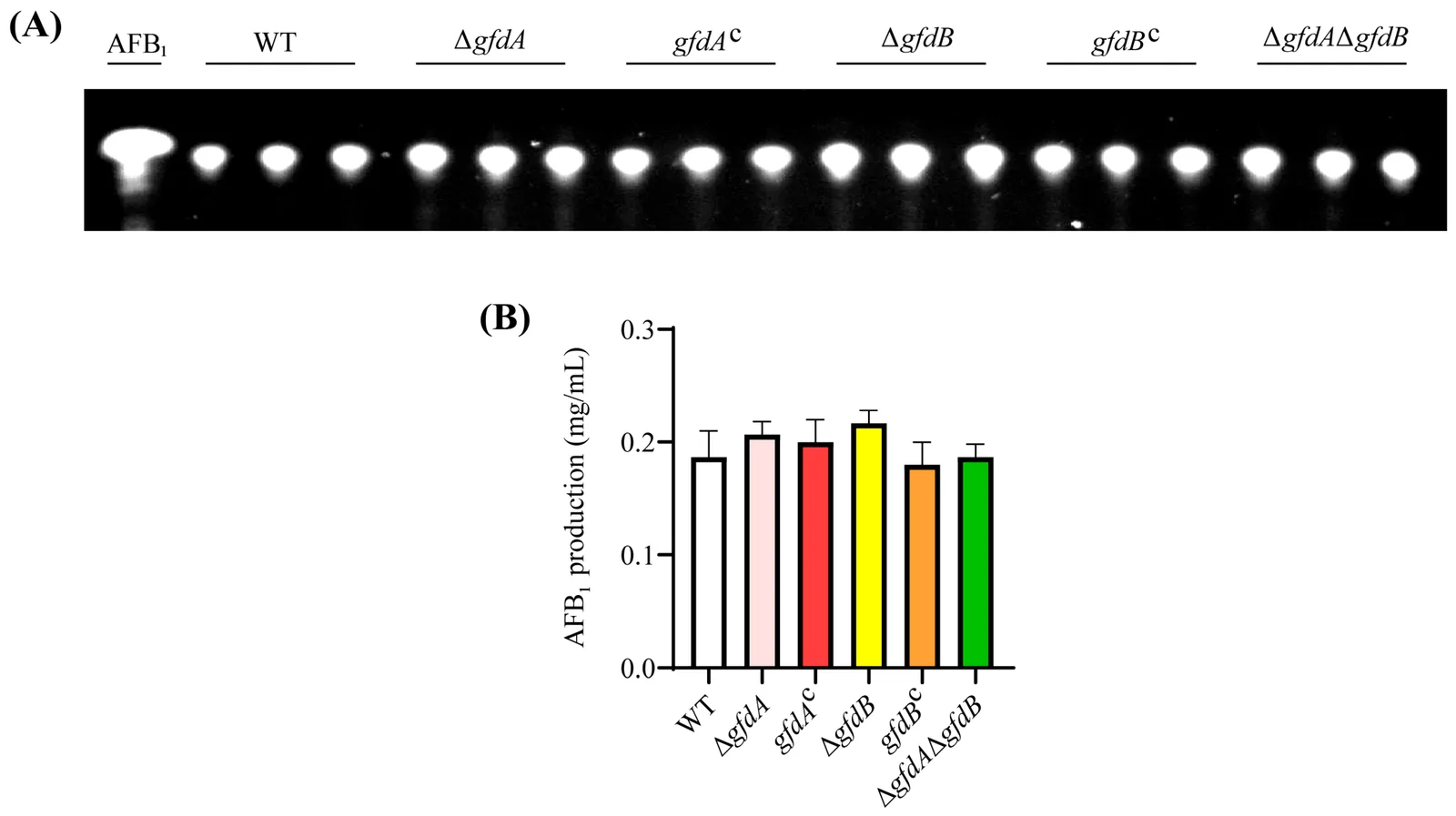
Analysis of aflatoxin B_1_ production in all strains. (**A**) TLC analysis of AFB_1_ production by the WT, Δ*gfdA*, Δ*gfdB*, *gfdA*^C^, *gfdB*^C^ and Δ*gfdA*Δ*gfdB* strains after 7 days at 29 °C in darkness. AFB_1_ indicates the standard. (**B**) Computation of AFB_1_ produced by the strains as in (**A**). Concentration of AFB_1_ standard was adjusted to 1 mg/mL; then the relative quantification of AFB_1_ was analyzed using GeneTools software 4.0.2.

In an attempt to analyze the influences of *gfdA* and *gfdB* genes on the production of AFB_1_, conidia suspensions were inoculated into liquid YES medium and cultured at 29 °C for 7 d. Then aflatoxins were extracted with organic solvent dichloromethane and detected with a thin-layer chromatography (TLC) method. As shown in Figure 5, the results showed that compared with WT, none of the tested strains exhibited a significant alteration in toxin content. It suggested that *gfdA* and *gfdB* genes did not make a notable contribution in the aflatoxin biosynthesis.

### 3.6. Pathogenicity of gfdA and gfdB Mutants on Seeds

**Figure 6:**
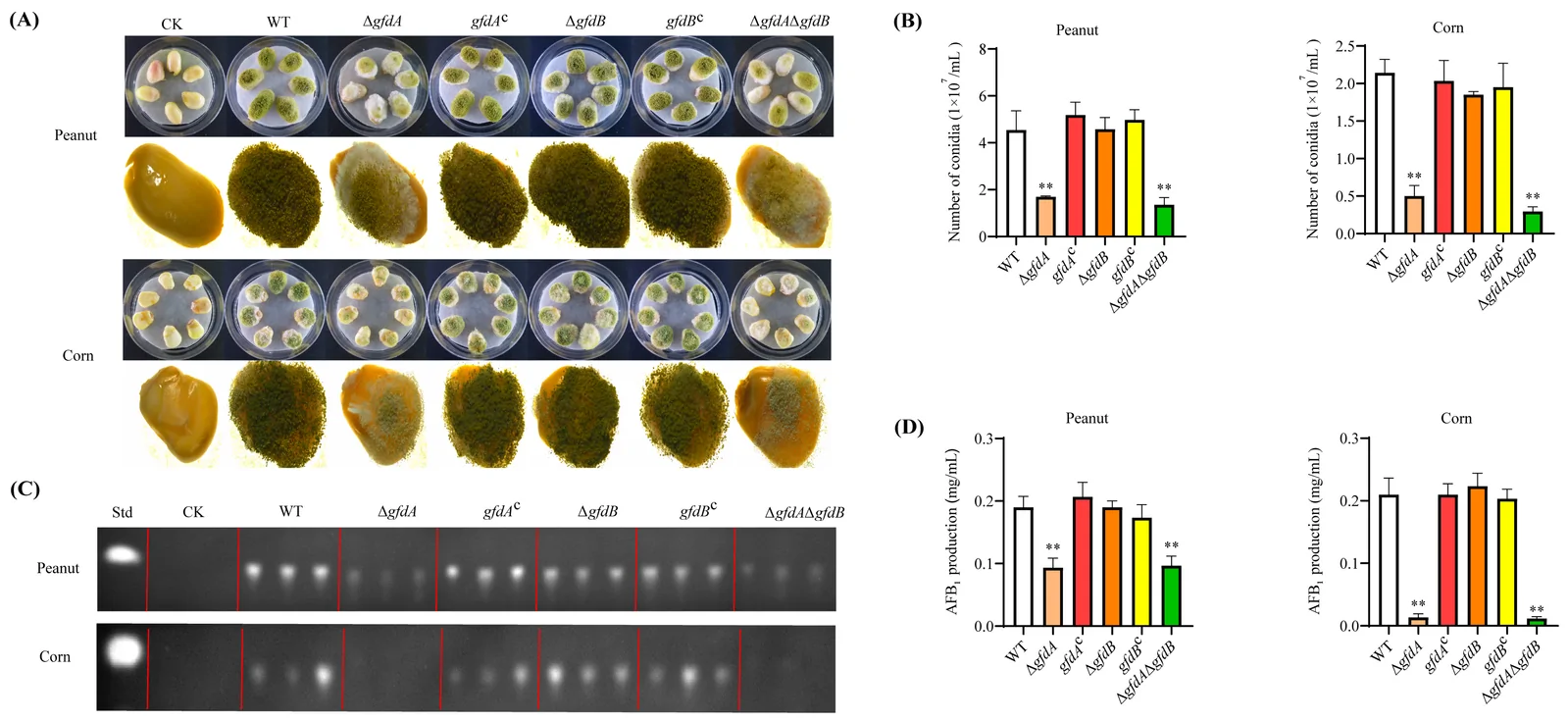
Pathogenicity of *gfdA* and *gfdB* mutants on peanut and corn. (**A**) Growth of all strains on peanut seeds or corn kernels after five days of inoculation. (**B**) Quantitative analysis of conidia produced on the infected peanut seeds and corn kernels as in (**A**). TLC analysis of AFB_1_ extracted from corn and peanut seeds infected by the strains in (**A**). AFB_1_ indicates the standard. (**D**) Quantitative analysis of AFB_1_ produced by the strains as in (**C**). The asterisks in (**B**,**D**) represent significant difference levels when Δ*gfdA*, Δ*gfdB* and Δ*gfdA*Δ*gfdB* strains were compared with WT (i.e., ** *p* ≤ 0.01). Standard deviation is indicated by error bars.

*A. flavus* and its secondary metabolites are widely present in nature and easily invade crops such as peanuts and corn, causing serious economic losses [35,36]. In the pathogenicity assay, the growth of the strains on the surface of peanuts and corn was recorded after 5 d at 29 °C. Preliminary observation showed that compared to the WT and complemented strains, the hyphal growth of Δ*gfdA* and Δ*gfdA*Δ*gfdB* strains was relatively sparse, while no significant difference was observed in Δ*gfdB* (Figure 6A). Then the conidia on the surface of peanuts and corn were washed and counted. The results showed that compared to the WT, the conidia yield of the Δ*gfdA* and Δ*gfdA*Δ*gfdB* strains decreased visibly, but the Δ*gfdB* strain had no effect (Figure 6B). Moreover, aflatoxin was extracted from the infected peanuts and corn and detected by the TLC assays (Figure 6C). It was observed that the amount of secondary metabolite AFB_1_ produced by Δ*gfdA* and Δ*gfdA*Δ*gfdB* double mutants was markedly reduced relative to the WT (Figure 6D). The above results showed that the deletion of *gfdA* gene can significantly reduce the ability of *A. flavus* to infect the seeds, meaning that *gfdA* has an important impact on the pathogenicity of *A. flavus* to the host, while *gfdB* gene does not.

### 3.7. Impacts of gfdA and gfdB Mutations on the Response of A. flavus to Osmotic Stress

**Figure 7:**
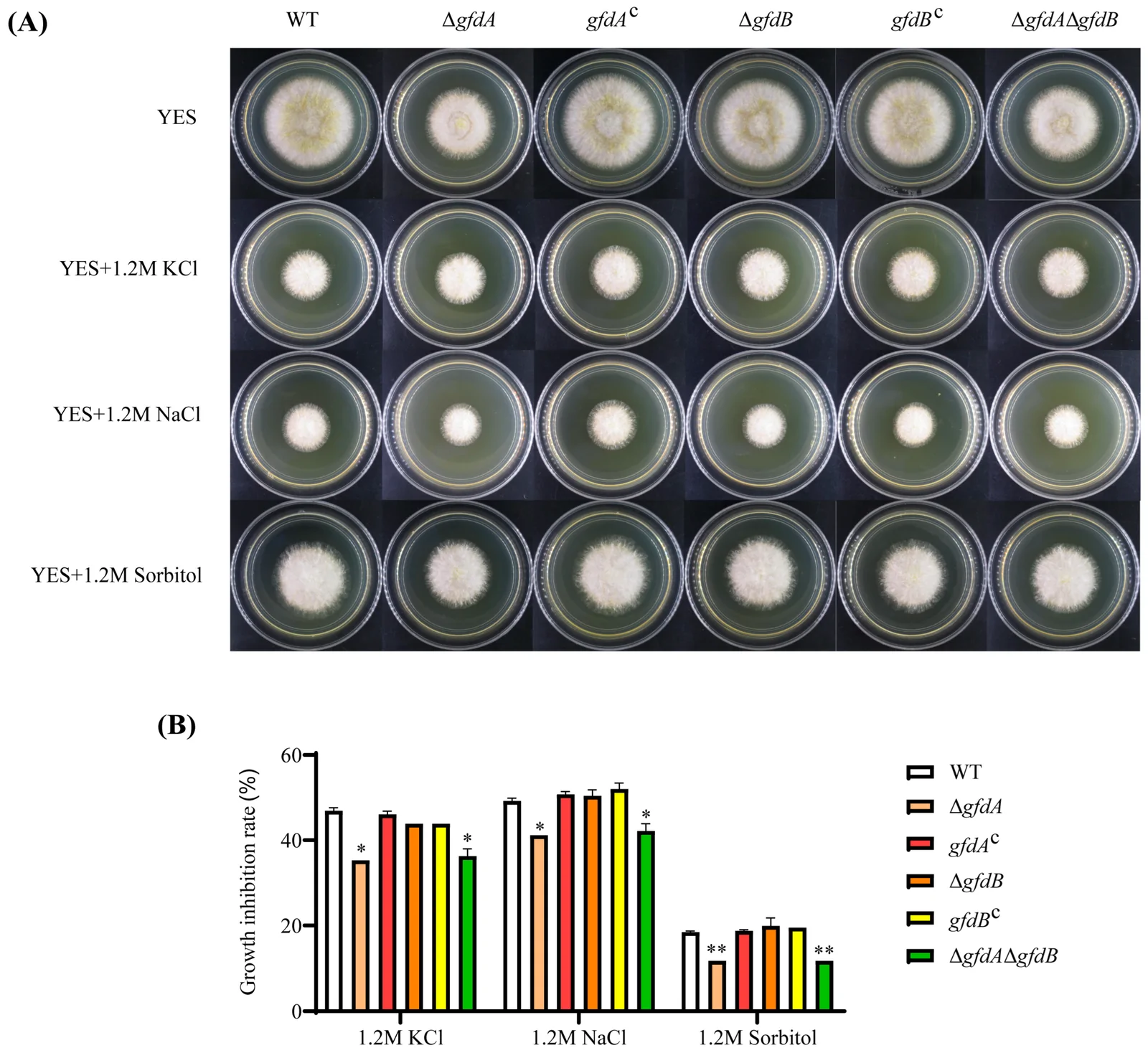
Effects of *gfdA* and *gfdB* on the response of *A. flavus* to osmotic stress. (**A**) Morphology of strains grown on YES medium containing osmotic reagents of 1.2 M KCl, 1.2 M NaCl or 1.2 M Sorbitol, then incubated at 37 °C for 3 days. (**B**) Growth inhibition rate of all strains under osmotic stress as in (**A**). The asterisks in (**B**) represent significant difference levels when Δ*gfdA*, Δ*gfdB* and Δ*gfdA*Δ*gfdB* strains were compared with WT (i.e., * *p* ≤ 0.05, ** *p* ≤ 0.01). Standard deviation is indicated by error bars.

Fungi possess unique growth and development mechanisms to adapt to environmental changes. Previous research has shown that glycerol, as an important compatibilizer in cells, can balance the changes under osmotic stress, and proteins encoded by the *gfdA* and *gfdB* genes are involved in the synthesis of glycerol in different organisms [37]. Therefore, we studied the functions of *gfdA* and *gfdB* genes in the response of *A. flavus* to osmotic stress. For osmotic stress assays, YES medium was used as the basal medium, supplemented with 1.2 M NaCl, KCl, or Sorbitol, respectively. The inhibition rate was calculated after cultivation for 3 d. The results showed that under osmotic stress, the growth inhibition rates of Δ*gfdA* and Δ*gfdA*Δ*gfdB* strains were noticeably decreased compared to WT and complemented strains, while that of Δ*gfdB* strains had no significant change (Figure 7). This indicated that the *gfdA* gene plays a role in the response of *A. flavus* to osmotic stress, but *gfdB* gene does not.

### 3.8. Effects of gfdA and gfdB Mutations on Glycerol Synthesis in A. flavus

**Figure 8:**
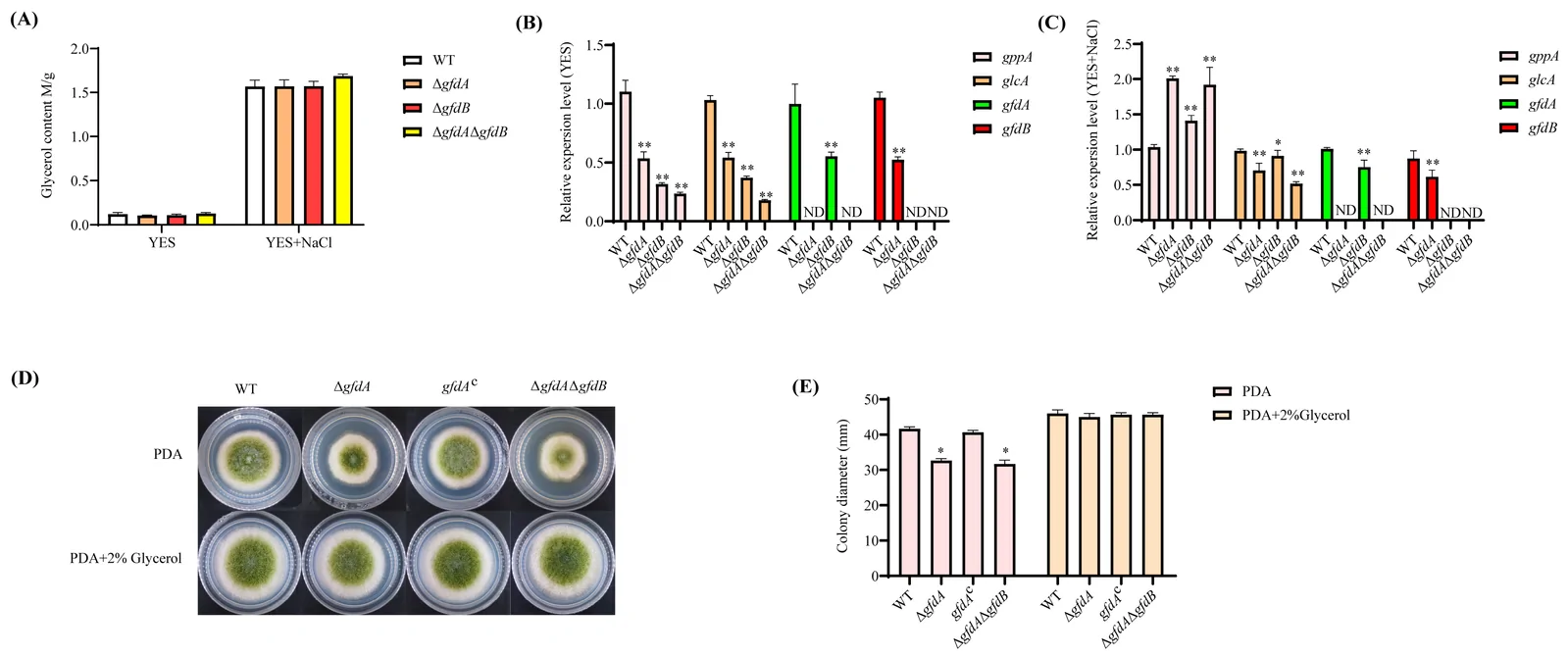
Effects of *gfdA* and *gfdB* mutations on glycerol synthesis in *A. flavus*. (**A**) Glycerol content of strains when treated with or without 1.2 M NaCl. (**B**) Relative expression levels of *gppA*, *glcA*, *gfdA* and *gfdB* in the tested strains when grown on YES medium. The primers used for RT-qPCR are shown in Table 3. (**C**) Relative expression levels of *gppA*, *glcA*, *gfdA* and *gfdB* in strains when grown on YES medium with 1.2 M NaCl. The primers used for RT-qPCR are shown in Table 3. (**D**) Morphology of *A. flavus* WT, Δ*gfdA*, *gfdA*^C^ and Δ*gfdA*Δ*gfdB* strains grown on PDA medium with or without 2% glycerol treatment. (**E**) Colony diameter of the strains in (**D**). The asterisks in (**B**,**C**,**E**) represent significant difference levels when Δ*gfdA*, Δ*gfdB* and Δ*gfdA*Δ*gfdB* strains were compared with WT (i.e., * *p* ≤ 0.05, ** *p* ≤ 0.01). Standard deviation is indicated by error bars.

According to previous reports [9,10], filamentous fungi have two glycerol synthesis pathways. One is from DHAP to G3P and then to glycerol; the other is from DHAP to DHA and then to glycerol. Glycerol-3-phosphate dehydrogenase encoded by *gfdA* and *gfdB* exactly catalyzes the conversion of DHAP to G3P and participates in the synthesis of glycerol. Therefore, we investigated whether *gfdA* and *gfdB* genes exert an influence on the synthesis of glycerol in *A. flavus*. Glycerol detection assays were performed as described above, and the results showed that the glycerol content of the Δ*gfdA*, Δ*gfdB* and Δ*gfdA*Δ*gfdB* strains did not change compared with WT, whether under normal conditions or under osmotic stress conditions (Figure 8A). It demonstrated that under normal and osmotic stress conditions, the absence of this synthetic pathway may be compensated by another. Meanwhile, it can also be found that the glycerol content of all tested strains under osmotic stress was obviously higher than that under normal conditions. And then, the expression levels of genes related to glycerol synthesis (*gppA*) and metabolism (*glcA*) were determined. qRT-PCR results showed that under normal conditions, *gppA* and *glcA* were significantly downregulated in all mutants (Δ*gfdA*, Δ*gfdB*, and Δ*gfdA*Δ*gfdB*). Under high osmotic stress, however, *gppA* was significantly upregulated, whereas *glcA* was downregulated in all mutants. Additionally, *gfdB* was downregulated in Δ*gfdA*, and *gfdA* was downregulated in Δ*gfdB* under both normal and high osmotic conditions (Figure 8B,C). To further verify if glycerol could make up for the growth defect caused by the deletion of *gfdA* gene, 2% glycerol was added to the PDA medium, and conidia were inoculated and cultured at 37 °C for 4 d in the dark (Figure 8D). The results shown that the addition of glycerol could almost completely restore the growth of the Δ*gfdA* and Δ*gfdA*Δ*gfdB* strains, indicating that glycerol indeed compensated for the growth defect caused by the deletion of the *gfdA* gene (Figure 8E).

## 4. Discussion

G3PDH binds to coenzyme I to catalyze the conversion of DHAP to G3P, and there are two homologous proteins, GPD1 and GPD2, in *S. cerevisiae* [15]. Using bioinformatics methods, we identified the homologous proteins GfdA and GfdB of *A. flavus*. The first step was to express these two proteins in vitro and determine their enzyme activities. It was verified that GfdA protein could combine with coenzyme NADH to catalyze the conversion of DHAP to G3P and exhibited G3PDH activity in vitro (Figure 2G). However, the corresponding activity of GfdB protein was not detected under the same conditions. Therefore, we aligned the protein sequences of GfdA and GfdB in *A. flavus* to determine the differences in the key domains and specific substrate-binding sites between the two proteins. It has been reported that the amino acid residues His94, Asn150, and Ser153 in mouse NAD^+^-dependent glycerol-3-phosphate dehydrogenase are highly conserved and function as specific substrate-binding sites, playing important roles in the catalytic reaction [38]. In *A. flavus, *sequence alignment revealed that His94 and Asn150 are highly conserved, whereas Ser153 in GfdB is replaced by Pro186. Thus, we speculated that the inactivation of the GfdB protein may be caused by amino acid substitutions at specific sites. Admittedly, this interpretation remains speculative and requires further experimental substantiation. In fact, studies in *A. fumigatus* have shown that *gfdA* gene deletion causes growth defects due to lack of G3P [23], and our study also demonstrated that Δ*gfdA* and Δ*gfdA*Δ*gfdB* strains have growth defects compared to WT, but the Δ*gfdB* strain has no growth inhibition. So we speculated that GfdA, rather than GfdB, is the key enzyme responsible for G3P synthesis. Combined with the obtained enzyme activity profiling data, we further hypothesized that the GfdB protein may be a partial functional redundancy with its paralog GfdA.

Although *gfdA* and *gfdB* are homologous genes in *A. flavus*, *gfdA* plays an important role in colony morphogenesis, conidia production and sclerotium formation, while *gfdB* does not show an obvious effect in these aspects. Specifically, compared to WT, the Δ*gfdA* mutant exhibited significantly reduced colony diameter, irregular marginal protrusions on colonies, a sharp decline in conidiation, and a severe defect in sclerotium formation. Studies in *A. nidulans* have demonstrated that deletion of *gfdA* results in severe growth defects and aberrant hyphal morphology [21], while the *gfdB* deletion mutant in *A. nidulans* showed no effect on hyphal growth and development [20], which is consistent with our findings. Notably, the double deletion mutant Δ*gfdA*Δ*gfdB* displayed nearly identical phenotypic traits to the *gfdA* single mutant. One possible reason is that GfdB protein may be functionally redundant compared to GfdA protein. An alternative explanation is that the GfdB protein exhibits distinct subcellular localization, which accounts for its functional divergence from GfdA. In *S. cerevisiae*, it has been reported that due to the different subcellular distribution of GPD1 and GPD2 proteins, they play distinct roles in oxidative stress [16]. It is worth noting that the Δ*gfdB* knockout mutant of *A. nidulans* exhibited pronounced sensitivity to oxidative stress (induced by hydrogen peroxide and tert-butyl hydroperoxide), cell wall integrity stress (induced by Congo red), and heavy metal stress [20]. Therefore, the potential role of GfdB in *A. flavus* under these additional environmental conditions merits further study.

As one of the most toxic secondary metabolites in nature, aflatoxin contamination poses a serious threat to food safety and human and animal health [39]. In this study, no effect of *gfdA* and *gfdB* mutations on aflatoxin biosynthesis was observed. However, in the seed infection experiment, in the process of infecting peanuts and corn with Δ*gfdA* and Δ*gfdA*Δ*gfdB* strains, the output of conidia and aflatoxin decreased obviously, compared to the WT. It is speculated that the reduction in conidia attached to the seed surface during infection may lead to the reduction in aflatoxin production. A similar infection experiment was carried out in *Pyricularia oryzae*, and it was discovered that the deletion of PoGpd1 or PoGpd2 would lead to reduced virulence of conidia or hyphae on rice, and the glycerol-3-phosphate shuttle is involved in virulence [40].

In fungi, glycerol biosynthesis has been proposed to play an important role in response to hyperosmotic stress. Indeed, in *S. cerevisiae*, GPD1 is responsible for most glycerol production under osmotic stimulus. *gpd1* knockout mutants produce very little glycerol, and *gpd1gpd2* double deletion strains did not produce glycerol anymore [17]. Nevertheless, in *A. nidulans*, inactivation of GpdA has a limited impact on glycerol synthesis. Its glycerol accumulation is not reduced during conidial germination, but intracellular glycerol levels are reduced to 60% of WT during growth under osmotic stress [21]. However, in *A. flavus*, the glycerol content of the Δ*gfdA*, Δ*gfdB*, Δ*gfdA*Δ*gfdB* strains showed nearly no change compared to WT under both normal and stress conditions. This result suggests that despite their high degree of protein sequence homology (84%), *gpd1* and *gfdA* fulfill distinct roles in the model yeast *S. cerevisiae* and the filamentous fungal genus *Aspergillus,* respectively. One possible explanation is that *Aspergillus* can use an alternative pathway (DHAP-DHP-glycerol) to synthesize glycerol, compared to *S. cerevisiae* which has only one main glycerol synthesis pathway (DHAP-G3P-glycerol). Interestingly, it was also found that the glycerol content in different strains under osmotic stress conditions was significantly higher than that under normal conditions, and the additional glycerol content under osmotic stress could restore the Δ*gfdA* growth defect. The possible reason is that the Δ*gfdA* strain cannot form sufficient G3P under normal conditions, but under the osmotic stress condition the Δ*gfdA* strain increases the glycerol content, and GlcA converts glycerol to G3P, thus restoring the growth defect. In fungi, osmotic stress responses are coordinated by a complex network of signal transduction pathways, including the cell wall integrity (CWI) pathway, the High-Osmolarity Glycerol (HOG) pathway, and the Target of Rapamycin (TOR) pathway, which exhibit extensive crosstalk to maintain cellular homeostasis [41]. In *A. fumigatus*, the TOR substrate SchA modulates Hog1 activity and affects glycerol accumulation under osmotic stress [42], supporting a link between TOR signaling and glycerol homeostasis. In *S. cerevisiae*, glycerol serves as a mediator of crosstalk between the HOG and CWI pathways [43]. Thus, the loss of Gfd proteins may be compensated via signaling-dependent regulation of alternative glycerol biosynthetic or catabolic enzymes, ensuring that glycerol levels remain stable under the conditions tested.

## 5. Conclusions

Overall, we preliminarily explored the main biological functions of the *gfdA* and *gfdB* genes encoding glycerol-3-phosphate dehydrogenase in *A. flavus* and confirmed that the *gfdA* gene played important roles in the growth and development of *A. flavus*, such as conidiation, sclerotium formation, etc., but *gfdB* did not play an obvious role in these aspects. Therefore, a more detailed understanding of the physiological functions of GfdB and the relationship between GfdA and GfdB remains to be fully elucidated in future investigations. Also, these findings advance our current understanding of glycerol metabolic pathways in *Aspergillus* and filamentous fungi.

## Figures and Tables

**Table 1:** *A. flavus* strains used in this study.

Strain	Description	Reference
*A. flavus* CA14 PTS	Δ*ku70*, Δ*pyrG*	Purchased from FGSC
Wild type	Δ*ku70*, Δ*pyrG*::*pyrG*	This lab preserves
Δ*gfdA*	Δ*ku70*, Δ*pyrG*, Δ*gfdA*::*pyrG*	This study
*gfdA* ^C^	Δ*ku70*, Δ*gfdA*::*pyrG*-*ptrA*, *pyrG*	This study
Δ*gfdB*	Δ*ku70*, Δ*pyrG*, Δ*gfdB*::*pyrG*	This study
*gfdB* ^C^	Δ*ku70*, Δ*gfdB*:*:pyrG*-*ptrA*, *pyrG*	This study
Δ*gfdA*Δ*gfdB*	Δ*ku70*, Δ*pyrG*, Δ*gfdA:*:*pyrG*, Δ*gfdB*::*ptrA*	This study

**Table 2:** *E.coil* strains and plasmid used in this study.

Strains and Plasmid	Function	Reference
*E.coil* DH5α	Used for amplifying recombinant plasmids	Purchased from TransGen Biotech
*E.coil* BL21	Used for protein induction expression	This lab preserves
pET-32a	Used to express strain construction	This lab preserves

**Table 3:** Primers used in this study.

Primer	Sequence (5′-3′)	Characteristics
*gfdA*-p1	CAGTACAATAAGTAGAACCCAA	For amplifying 5′UTR of ∆*gfdA*
*gfdA*-p3	GGGTGAAGAGCATTGTTTGAGGCCTGCGAATGAGACACGAA	
*gfdA*-p6	GCATCAGTGCCTCCTCTCAGACATAGGACCTGATGCTTTGT	For amplifying 3′UTR of ∆*gfdA*
*gfdA*-p8	CTTAGATGTTGGGATTGC	
*gfdA*-p2	CTGCCGATAGTAGATAAGATG	For fusion PCR of ∆*gfdA*
*gfdA*-p7	CAAGAAGTAGCCTGGGTG	
*gfdA*-p9	TCCAGCAACGGACACTCT	For amplifying ORF of ∆*gfdA*
*gfdA*-p10	AATCAAGTCGGCAACACC	
*gfdA*-C1	AAGCAGTACAATAAGTAGAA	For amplifying 5′UTR and ORF of ∆*gfdA*
*gfdA*-C2	CGAGGTGCCGTAAAGCACTAATTACCGCTCAATATAAGAAGGGATA	
*ptrA*-F	TTAGTGCTTTACGGCACCTCG	For amplifying *ptrA*
*ptrA*-R	ACTTTATCCGCCTCCATCCAG	
*gfdA*-*pyrG*-F	CTGGATGGAGGCGGATAAAGTGCCTCAAACAATGCTCTTCACCC	For amplifying *pyrG* of *gfdA*^C^
*gfdA*-*pyrG*-R	GTCTGAGAGGAGGCACTGATGC	
*gfdA*-O1	CAACGGTAGGTTTGATTTA	For fusion PCR of *gfdA*^C^
*gfdA*-O2	GTATTTGCGAGCGTAGTC	
*gfdB*-P1	AGCCTTGACCTATTTACA	For amplifying 5′UTR of ∆*gfdB*
*gfdB*-P3	GGGTGAAGAGCATTGTTTGAGGCTTTATGCTATTTGGATGG	
*gfdB*-P6	GCATCAGTGCCTCCTCTCAGACGCGCTGTTTCTGTGAACT	For amplifying 3′UTR of ∆*gfdB*
*gfdB*-P8	CGCAAACCCATTCTTCTA	
*gfdB*-P2	GCCCTTAGACGTGGACGAT	For fusion PCR of ∆*gfdB*
*gfdB*-P7	GGTAACTACTAAACGCCACTT	
*gfdB*-C1	CCCGACAGATAGACCAAC	For amplifying 5′UTR and ORF of ∆*gfdB*
*gfdB*-C2	CGAGGTGCCGTAAAGCACTAATCATAGGCGAGACTCACCAT	
*gfdB*-*pyrG*-F	CTGGATGGAGGCGGATAAAGTGCCTCAAACAATGCTCTTCACCC	For amplifying *pyrG* of *gfdB^C^*
*gfdB*-*pyrG*-R	GTCTGAGAGGAGGCACTGATGC	
*gfdB*-P9	CACCCAATACTAACCACCAA	For amplifying ORF of ∆*gfdB*
*gfdB*-P10	CAGAACAAGATGCGACCA	
*pyrG*-F	GCCTCAAACAATGCTCTTCACCC	For amplifying *pyrG*
*pyrG*-R	GTCTGAGAGGAGGCACTGATGC	
*pyrG*-P1	ATCGGCAATACCGTCCAGAAGC	For screening *pyrG*
*pyrG*-P2	CAGGAGTTCTCGGGTTGTCG	
*gfdAgfdB*-P1	GATGCGAACTGCTATTGACCTG	For amplifying *gfdB* (5′UTR) of ∆*gfdA*∆*gfdB*
*gfdAgfdB*-P2	CGAGGTGCCGTAAAGCACTAACCTTGCCTGCACAACTGTCTTA	
*gfdAgfdB*-P3	CTGGATGGAGGCGGATAAAGTGTCCTCAGTCCCTTTGCTATCC	For amplifying *gfdB* (3′UTR) of ∆*gfdA*∆*gfdB*
*gfdAgfdB*-P4	TGTAACCTACCATATCAGCGTGT	
*gfdAgfdB*-O1	CCCATATTATCATATCTGTCTCCG	For fusion PCR of ∆*gfdA*∆*gfdB*
*gfdAgfdB*-O2	TCAGGTAACTACTAAACGCCACT	
*ptrA*-P1	CAACACTATCAACGCTCCTGTC	For screening *ptrA*
*ptrA*-P2	ATTGTCGGTTGGTTTGGGAACT	
*abaA*-F	CTTCTTTCTTGTTCTCAGCTG	qRT-PCR validation of genes associated with chlamydospore formation
*abaA*-R	TAGGCGACCCCGTTCC	
*brlA*-F	ATCCTTTCCGTTCTCTGGC	qRT-PCR validation of genes associated with chlamydospore formation
*brlA*-R	AGATTGTCAGCTCTGGTGC	
*nsdC*-F	GCCAGACTTGCCAATCAC	Internal reference gene for qRT-PCR
*nsdC*-R	CATCCACCTTGCCCTTTA	
*nsdD*-F	GGACTTGCGGGTCGTGCTA	For screening ∆*gfdA* for qRT-PCR
*nsdD*-R	AGAACGCTGGGTCTGGTGC	
*Actin*-F	ACGGTGTCGTCACAAACTGG	For screening ∆*gfdB* for qRT-PCR
*Actin*-R	CGGTTGGACTTAGGGTTGATAG	
*gfdA*-F	GGCGTTGCCCTCCGAGTAT	For amplifying 5′UTR of ∆*gfdA*
*gfdA*-R	GCTTTGGCGTTGTCACCC	
*gfdB*-F	TATCGTGCGTGAAGGGCGTAG	For amplifying 3′UTR of ∆*gfdA*
*gfdB*-R	TCCATCGGCGGCGTATCA	
*gppA*-F	CTTTATCGAAGGCCGCATCCC	qRT-PCR validation of genes related to glycerol anabolic metabolism
*gppA*-R	CGACATCCTCCGCGACAACA	
*glcA*-F	GCAGCAACGGTTCCAGACAG	
*glcA*-R	CGACGGCGAGTTCCGACAAT	
Lic-*gfdA*-F	GCCATGGCTGATATCGGATCCCTGGAAGTTCTGTTCCAGGGGCCCATGCTGTCCCCAGTACATCTTCTATCTC	For cloning the *gfdA* gene
Lic-*gfdA*-R	GGTGGTGGTGGTGCTCGAGTCACAATTTGGCCAAGACTGCCT	
Lic-*gfdB*-F	AATGGGTCGCGGATCCCTGGAAGTTCTGTTCCAGGGGCCCATGCCACAGCAAAAGAAACAACACA	For cloning the *gfdB* gene
Lic-*gfdB*-R	GGTGGTGGTGGTGGTGCTCGAGCTAACCGAACCTCTTGTCCAGCAACTTT	

## Data Availability

All data generated or analyzed during this study are included in this published article.
